# Phytochemical analysis and anticancer activity of *Falcaria vulgaris* Bernh growing in Moghan plain, northwest of Iran

**DOI:** 10.1186/s12906-021-03464-2

**Published:** 2021-12-05

**Authors:** Kamran Hosseini, Sanaz Jasori, Abbas Delazar, Parina Asgharian, Vahideh Tarhriz

**Affiliations:** 1 Molecular Medicine Research Center, Biomedicine Institute, Tabriz University of Medical Sciences, Tabriz, Iran; 2 Department of Molecular Medicine, Faculty of Advanced Medical Sciences and Technologies, Shiraz University of Medical Sciences, Shiraz, Iran; 3 Biotechnology Research Center, Tabriz University of Medical Sciences, Tabriz, Iran; 4 Drug Applied research Center, Tabriz University of Medical Sciences, Tabriz, Iran; 5 Department of Pharmacognosy, Faculty of Pharmacy, Tabriz University of Medical Sciences, Tabriz, Iran

**Keywords:** *Falcaria vulgaris* Bernh, Cancerous cells, GC-MS, Cytotoxicity, Apoptosis, Volatile compounds

## Abstract

**Background:**

*Falcaria vulgaris* Bernh among the most important member of *Apiaceae* family has been used for medical investigation in Iran and some regions in the world. This plant possesses a range of coumarin and flavonoids compounds that have many therapeutic properties such as gastrointestinal and liver diseases, skin ulcers, gastric ulcers, and intestinal inflammation. It has also been found that these compounds lead to cytotoxic effects.

**Objective:**

This study contains concentrates on the cytotoxic effect and induction of apoptosis on cancerous cells (SW-872) through various extracts and essential oil of *Falcaria vulgaris* Bernh. It considers the volatile compounds of effective samples.

**Methods:**

The shoot of the plant was extracted by the Soxhlet apparatus and its essential oil was taken by the Clevenger apparatus. The cytotoxicity of the samples was evaluated by the MTT method and the mechanism of cancer cell death by flow cytometry and finally, the volatile compounds of essential oils and effective extracts were identified by GC-MS.

**Results:**

The results demonstrated that *n*-Hexane extract and 40% VLC fraction had the greatest cytotoxic effect on SW-872 cells. While, the most abundant volatile compounds in essential oil and 40% VLC fraction of *n*-Hexane extract were terpenoid compounds like (+) spathulenol and caryophyllene oxide, in *n*-Hexane extract tetradecan, and spathulenol were the most, respectively.

**Conclusion:**

The fraction of 40% *n*-Hexane was in a concentration-dependent manner and significantly with controlling cells inhibited the growth of cancer cells. A plausible explanation could be made to account for this effect. This inhibition was made through induction of apoptosis and due to the presence of effective volatile compounds such as terpenoids and non-terpenoids which could be considered as valuable natural sources for the isolation of anti-cancer compounds.

## Introduction

Cancer is a disease in which the growth and proliferation of cells increase uncontrollably and can spread to other parts of the body [1] In 2020 in the United States, about 1.8 million new cases of cancer were reported, of which about 606 died [2]. So far, effective therapies for the prevention and treatment of cancer, including immunotherapy, hormone therapy, radiotherapy, chemotherapy and surgery, have been reported [3]. Despite extensive research into cancer and the discovery of related treatments, it is still considered a scary disease [4]. On the other hand, after years of progress in the field of health, today developing countries are involved in the fight against deadly cancers [3]. At present, nonspecific function, drug resistance and severe side effects are among the main problems of chemotherapy drugs. Therefore, there is an urgent need for extensive studies to find alternative and complementary therapies [5]. Using high-performance screening, researchers have found that a variety of natural products, such as herbs, can be used as therapeutic agents in chemoprevention [6]. Scientific information on the safety and effectiveness of herbal remedies has shown that these plants have good anti-cancer activity and most of them are currently under clinical trial [7–9].

The compounds in plants play a key role in killing malignant cancer cells through the apoptotic pathway [10, 11]. Apoptosis or programmed cell death is a defense mechanism that occurs due to molecular events and changes in cell morphology such as blabbing, cell shrinkage, nuclear fragmentation, chromatin condensation, and so on [12]. Therefore, many researchers have tried to discover therapeutic agents to stimulate malignant cancer cells by apoptosis, to offer a new treatment strategy [13]. In this regard, many studies have reported the therapeutic properties of plants of the *Apiaceae* family, especially the genus *F. vulgaris* (*Falcaria vulgaris* Bernh) [14–16]. The plants of this family are widely used in traditional Iranian medicine and have antimicrobial effects [14], treatment of gastrointestinal diseases [17, 18], skin diseases [19], anti-fertility [20], analgesia [21, 22], treatment of heart diseases [23], etc.

Among the effective compounds of plants of this family is Spathulenol, Carvacrol, Germacrene-B, α-pinene, α-Terpinyl acetate and β-caryophyllene [24–28]. The plants of this family are mainly distributed in the northern, northwestern and western regions of Iran and grow mostly in East Azerbaijan province [29]. The aim of the present study was to investigate the cytotoxic effects of *F. vulgaris* on SW-872 (skin cancer) cancer cell line.

## Materials and methods

### Chemicals

Substances such as *n*-Hexane (*n*-Hex), dichloromethane, methanol and ethyl acetate (Caledon Canada), RPMI1640, FBS, Penicilin/Streptomycin (Gibco™ Invitrogen, USA) and MTT (3-(4,5-Dimethylthiazol-2-yl)-2,5-diphenyl tetrazolium bromide) (Sigma-Aldrich, USA) were purchased.

### Plant collection and drying

*F. vulgaris* plants were collected in the spring of 2016 from Moghan region located in Ardabil province (39°22′20.6″N 47°44′56.9″E). The plant was identified by Dr. Fatemeh Ebrahimi at the herbarium of Tabriz University of Medical Sciences, Tabriz, Iran (Code number: TBZ-FPH 178). After washing the collected samples, the shoots of *F. vulgaris* were completely dried in the open air and laboratory conditions and then ground into a fine powder by an electric mill.

### Extraction and fractionation

First, 200 g of *F. vulgaris* plant powder was weighed and after loading in a suitable filter paper, it was placed in a 1-l Soxhlet apparatus and extracted with *n*-Hex, dichloromethane (DCM) and methanol (MeOH) solvents, respectively, for 72 h. The extracts were then dried completely by rotary evaporator at 45 °C under low air pressure and finally weighed. Effective non-polar extracts were fractionated using VLC method with the stationary phase of silica gel, *n*-Hex solvents and increasing percentages of ethyl acetate. Another type of preparative thin-layer chromatography, or PTLC, is called the VLC technique, in which a separation process is performed using silica gel or aluminum hydroxide attached to a Buchner funnel. After each fractionation, the chromatographic column dries because re-running the dried PTLC plates increases the separation efficiency. This method can be considered as an alternative to conventional chromatographic methods and can be used to isolate compounds such as steroids, flavonoids, alkaloids, glycosides, xanthones, and diterpenes and so on [30]. Non-polar extracts can be fractionated using VLC method in several steps. First, we put a filter paper on the funnel connected to Erlenn Buchner, and then added silica gel to it. Another filter paper was placed on the surface of the silica gel and fix it. After that, about 150 mL of methanol, 150 mL of ethyl acetated, and 150 mL of 10% hexane were passed through silica gel, respectively. Dissolve 2 g of the plant sample in about 7 to 8 cc of 10% ethyl acetate and spread it on a smooth silica gel surface. We passed 10, 20, 40, 60, 80 and 100% of ethyl acetate solvents in *n*-Hex from the bottom to the top of the column, respectively, and at the end, we passed pure methanol. Following this method, six fractions were obtained which dried using a rotary evaporator [30, 31]. Extracts and fractions were stored frozen until use.

### Essence extraction

The choice of essential oil extraction method depends on the type and condition of the plant, the active ingredients available and the purity of the final product. The water distillation process was used on dry plants to prevent the destruction of compounds due to the high boiling temperature of the water. In essential oil distillation, 100 g of plant shoot powder with 150 mL of distilled water and 50 mL of glycerin were poured into a 1-l round bottom balloon. The balloon was then connected to a clevenger device and placed in a mantel heater to provide the necessary heat to penetrate the plant tissues. Glycerin also helps to extract essential oils from plant powder by increasing the permeability of plant cell walls. A Clevenger device with a functional design is the most suitable tool for cooling the vapors emitted from the sample and extracting the resulting essential oil. The essential oil extraction process was performed for 4 h according to the British Pharmacopeia method. Finally, the essential oil was then collected in a microtube and stored in a refrigerator.

### Identification of volatile compounds in non-polar samples

Gas chromatography-mass spectrometry (GC-MS) was used to identify compounds in essential oils and extracts of *n*-Hex and its VLC fraction of *n*-Hex extract. GC-MS analysis was performed on a Shimadzu GC-MS QP 5050A gas chromatograph-mass spectrometer fitted with a fused capillary column DB-1 (60 m, 0.25 mm id, film thickness 0.25 μm) [32]. Helium was utilized as the carrier gas, at a flow rate of 1 mL/min.

### Cell culture

SW-872 (Human liposarcoma cell line) and HFF-2 (Normal human skin fibroblasts) cells were obtained from the Pasteur Institute of Iran and grown in RPMI-1640 medium containing 10% FBS supplemented with 1% antibiotic (penicillin/streptomycin) at 37 °C in a humidified, 5% CO_2_ incubator.

### Preparation of plant samples

10 mg of each dried extract was carefully weighed and completely dissolved in separate microtubes in 100 μl DMSO; then the contents of each microtube were brought to a volume of 1 mL using 900 μl of complete culture medium. Due to the concentration of active herbal substances in the fractions of each extract, their diluted samples were prepared with 0.1 concentration of total extracts. Therefore, the exact amount of 1 mg of each fraction was dissolved in 10 μl of DMSO and brought to a volume of 1 mL.

### MTT assays

MTT assay was used for determination of substances toxicity on cell lines and evaluating of IC_50_ Cell suspension with a concentration of 3 × 10^4^ cells per mL was prepared and inoculated in 96-well microplates for 24 h. The appropriate amount of extracts and fractions were added to control and test wells and the plates were incubated for 24 and 48 h. Moreover, 20 μl of MTT solution was added to each well and incubated for 3 h. The supernatant was replaced with 150 μl of DMSO and dissolved the formazan crystals. Finally, it was measured by the ELISA plate reader at 570 nm (Anthos, Austria).

### Detection of apoptosis by flow cytometry

At this stage, the Annexin-V-Fluos Staining kit was used, which contains both AnnexinV material (to bind to the phospholipid molecule) and PI dye, which makes it possible to identify and clean apoptotic cells from necrotic cells. For flow cytometric testing, SW-872 cells were distributed in 6 plates so that each well contained 2 × 10^5^ cells. After 24 h of incubation, the cells were treated with IC_50_ concentrations of fractions in pairs. Then the cells were isolated from the bottom of the well and transferred to separate microtubes. After centrifugation, the supernatant was removed and the cells were washed with 500 μl of PBS and centrifuged again. The washed cells of each microtube were slowly dispersed in 100 μl of diluted Annexin V Binding buffer and 5 μl of Annexin solution and PI dye were added to each microtube and incubated in the dark for 30 min at room temperature. Then the cells were centrifuged and after removing the supernatant, they were dispersed in 200 μL of Annexin V Binding buffer. Finally, the absorption intensity and fluorescence of the samples were read using a flow cytometer at 488 wave lengths.

### Statistical analysis

In the present study, all experiments were repeated at least twice and the results were obtained as mean ± standard deviation by descriptive statistics. Statistical analyzes were performed using Graph pad prism 8 software. For comparison between groups, ANOVA and Tukey post hoc test were used and the minimum significance level was considered *p* < 0.05.

## Results

### Amounts of extracts and fractions

The amount of extracts obtained from one extract of 200 g of aerial part powder of *F. vulgaris* plant using Soxhlet method and solvents of *n*-Hex, DCM and MeOH is shown in Table 1. In addition, the results of fractionation of 2 g of *n*-Hex extract by VLC method with *n*-Hex solvents and increasing percentages of ethyl acetate and silica gel stationary phase are shown in Table 1.

**Table 1:** Weighing results of extracts obtained from *F. vulgaris* and its fractionation of *n*-Hex and DCM extracts

MeOH		DCM		*n*-Hex		Extract
9.04		2.12		1.35		Weight of plant extract (g/100 g of extract)
**100%**	**80%**	**60%**	**40%**	**20%**	**10%**	Fractions
4.35	16.25	8.6	12.35	20.05	4.55	Weight of *n*-Hex extract fraction (g/100 g of extract)
11.95	13.15	10.20	2.25	1.30	0	Weight of DCM extract fraction (g/100 g of extract)

### Results of cytotoxicity on SW-872 cell line

**Fig. 1:**
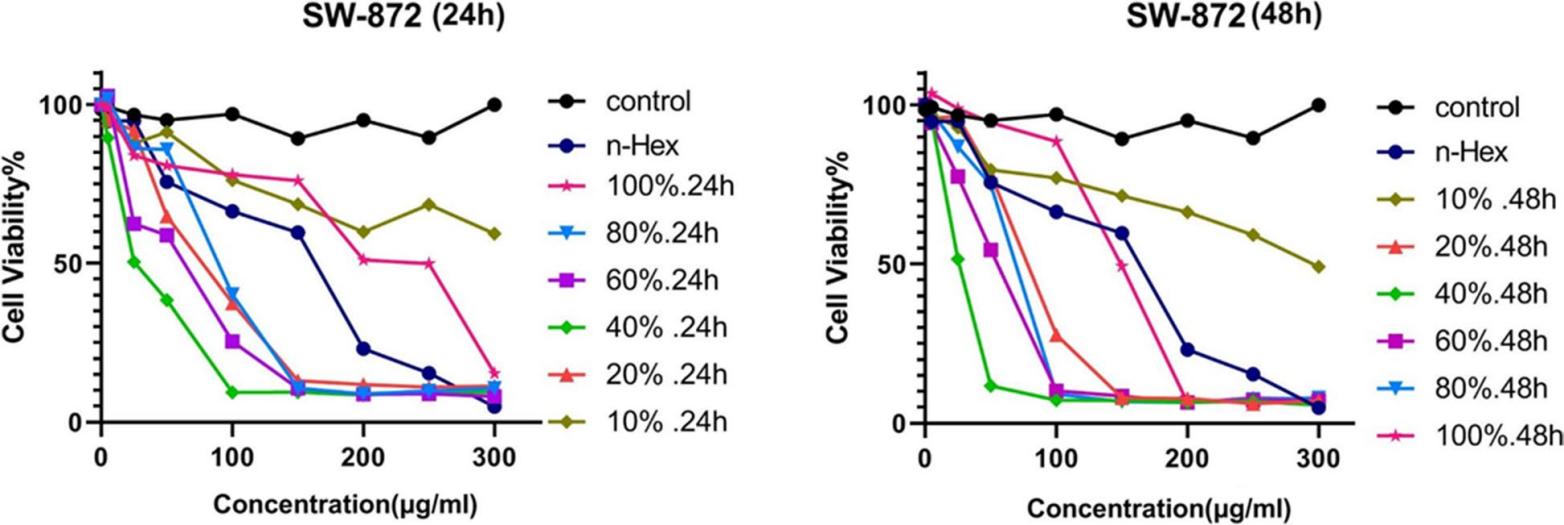
Determination of viability of SW-872 cells treated with different concentrations of *n*-Hex extract fractions of *F. vulgaris* using MTT method at 24 h and 48 h

The results of MTT testing of *n*-Hex, DCM and MeOH extracts on SW-872 cells at 24 and 48 h. According to the results, we observed the remarkable cytotoxicity of *n*-Hex extract on SW-872 cells, compared to other extracts and DMSO control; therefore, in the next step, *n*-Hex fractions were tested by MTT. The results of this test at 24 and 48 h are shown in Table 2 and Fig. 1.

**Table 2:** Cytotoxic effect of *n*-Hex, DCM and MeOH extracts and fractions of *n*-Hex extract of *F. vulgaris* on SW-872 cells

IC_50_(μg/ mL)		Sample	
48 h	24 h		
108.70 ± 5.72	138.40 ± 3.89	*n*-Hex extract	
163.70 ± 6.92	209 ± 24.53	DCM extract	
> 800	> 800	MeOH extract	
> 300	> 300	10% Fraction	*n*-Hex extract fractions
68.16 ± 3.17	74.22 ± 5.73	20% Fraction	
24.42 ± 0.09	32.04 ± 1.27	40% Fraction	
48.92 ± 13.95	48.13 ± 2.73	60% Fraction	
63.34 ± 0.01	85.36 ± 3.54	80% Fraction	
146.40 ± 12.02	168.40 ± 31.75	100% Fraction

**Fig. 2:**
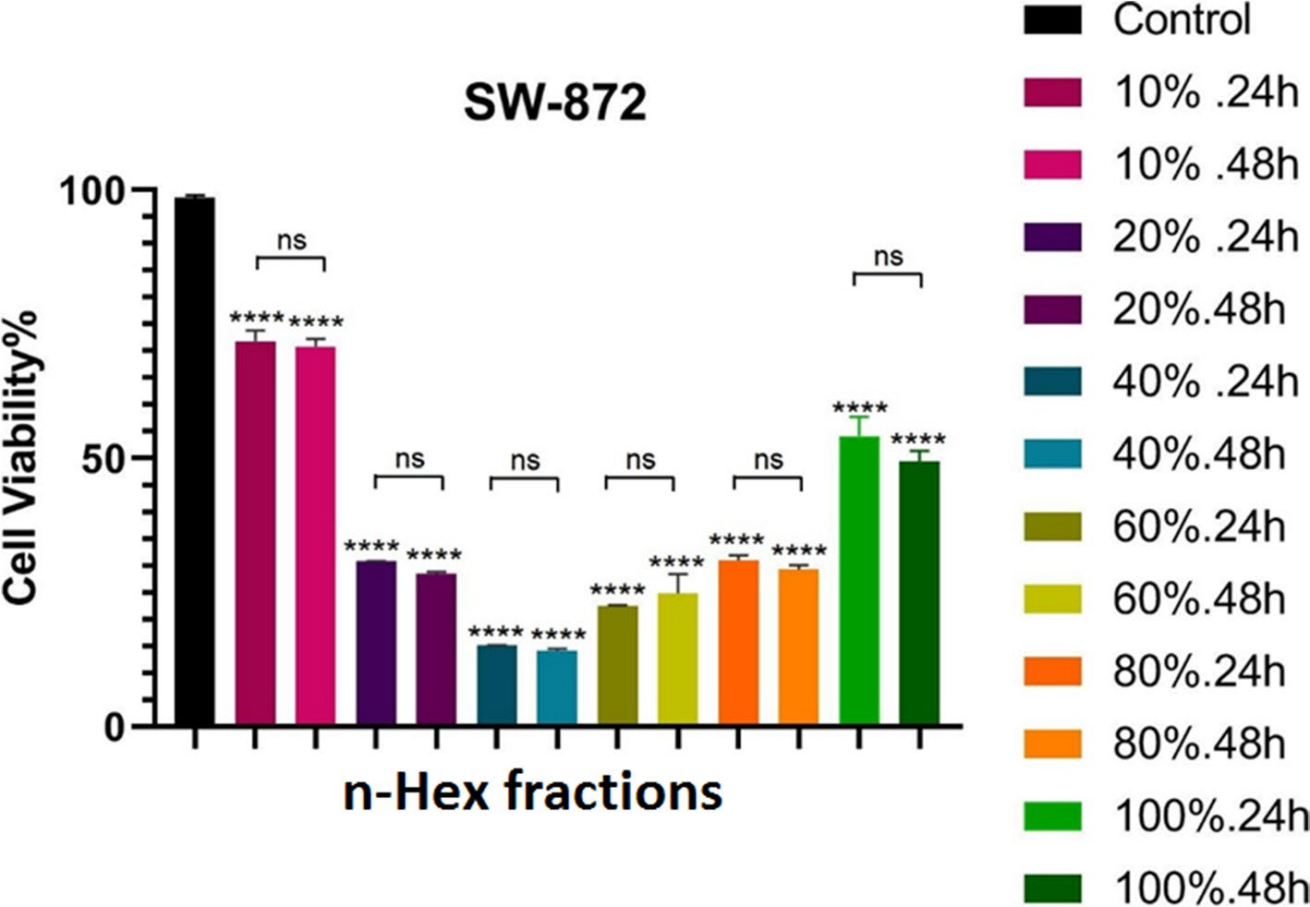
Statistical comparison of viability of SW-872 cells treated with *n*-Hex extract of *F. vulgaris* compared to the control group, using MTT method

To compare the cytotoxic effect of different plant samples at 24 and 48 h, Two Way ANOVA test and TUKEY post hoc test were performed to compare the effect of different groups together with DMSO control. The results show that all samples had a significant cytotoxic effect compared to DMSO control. In statistical tests, the significance level was defined as ns (*p* > 0.05), * (*p* < 0.05), ** (*P* < 0.01), *** (*p* < 0.001), **** (*P* < 0.0001) Becomes. A significant comparison of the effect of different samples with controls is presented in Fig. 2.

### Results of cytotoxicity on HFF-2 cells

To investigate the effect of cytotoxicity of *F. vulgaris* extracts and fractions on non-cancerous cells, MTT assay was performed on normal HFF-2 cell lines. Table 3 shows the IC50 results of 24- and 48-h treatments on these cells.

**Table 3:** Cytotoxic effect of *n*-Hex, DCM and MeOH extracts and fractions of *n*-Hex extract of *F. vulgaris* on HFF-2 cells

IC_50_(μg/ mL)		Sample	
48 h	24 h		
46.11 ± 8.41	122.70 ± 19.58	*n*-Hex extract	
60.68 ± 10.34	76.26 ± 16.38	DCM extract	
> 800	> 800	MeOH extract	
> 300	> 300	10% Fraction	*n*-Hex extract fractions
47.54 ± 3.73	40.95 ± 1.36	20% Fraction	
33.84 ± 0.46	49.30 ± 0.49	40% Fraction	
29.55 ± 0.59	11.38 ± 2.34	60% Fraction	
49.27 ± 1.49	55.26 ± 2.67	80% Fraction	
48.38 ± 5.47	114.10 ± 8.34	100% Fraction	

### Apoptosis test results in SW-872 cells

**Fig. 3:**
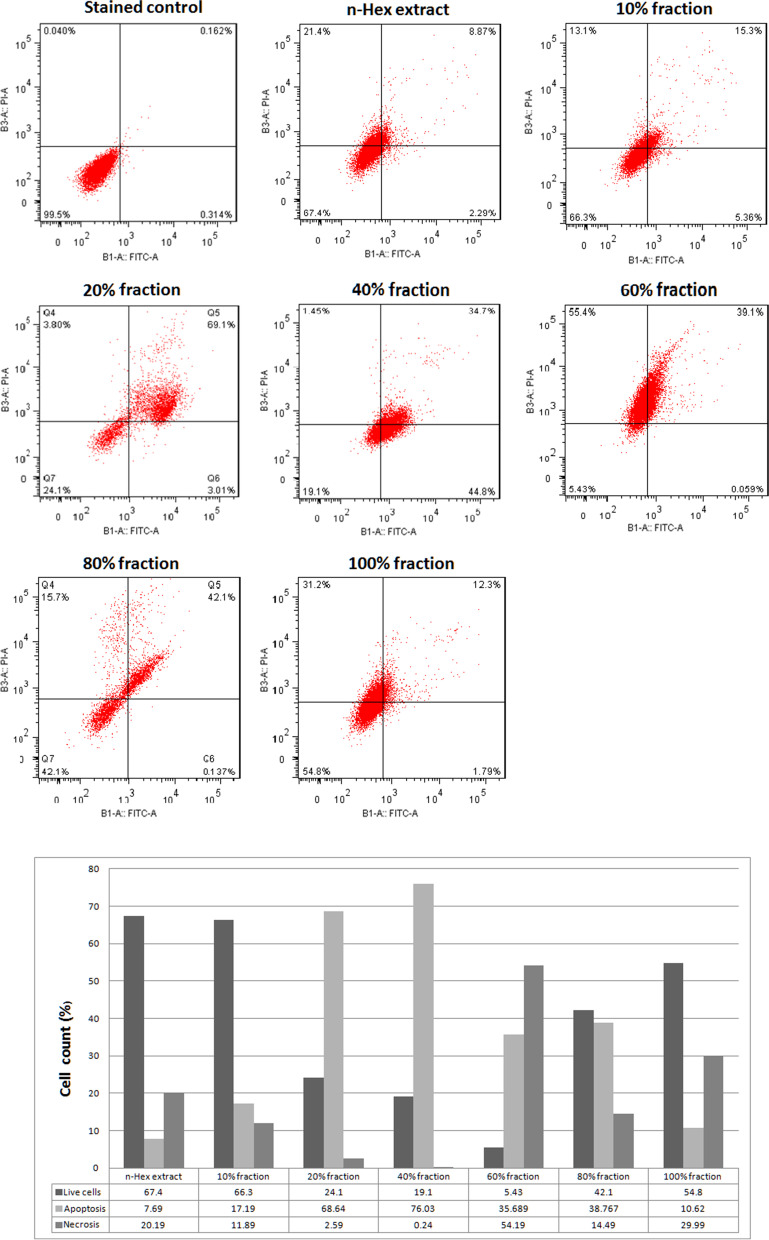
**a** Apoptosis rate of cells treated with *n*-Hex extract fractions on SW-872 cells. **b** Comparison of the mechanism of SW-872-induced cell death with *n*-Hex extract and its fractions

Flow cytometry test was performed to evaluate the mechanism of cell death induced by DCM extract fractions on SW-872 cells and the results are shown in Fig. 3. The results showed that the 20 and 40% fractions of *n*-Hex extract have the highest induction of apoptosis on SW-872 cells and have a suitable cell death mechanism.

### Identification of compounds in essential oil using gas chromatography-mass spectrometry

Using the GC-MS, the compounds in the volatile oil of *F. vulgaris* were identified, which are shown in Table 4 with specifications such as inhibition time, percentage of constituents and KI.

**Table 4:** Specifications of volatile compounds identified in essential oils using GC-MS

No	Name	Rt	Area%	KI
1	Octanal	18.62	3.93	981
2	o-Cymene	20.26	0.6	1013
3	2-Nonanone	23.44	0.58	1072
4	Nonanal	24.06	0.64	1084
5	2-Decenal	32.11	2.48	1240
6	Copaen	38.83	1.45	1380
7	trans-Caryophyllene	40.78	3.57	1422
8	beta.-copaen-4 .alpha.-ol	42.26	1.36	1455
9	Naphthalene	43.10	2.56	1474
10	germacrene d	43.37	1.29	1480
11	Delta-Cadinene	45.02	4.49	1519
12	caryophyllene oxide	46.14	18.3	1545
13	1,5-epoxysalvial-4 (14)-ene	46.81	2.63	1561
14	Isospathuleno	47.08	4.31	1567
15	(+) spathulenol	47.30	33.8	1573
16	Salival-4 (14)-en-1-one	47.82	3.23	1585
17	Epiglobulol	48.16	1.85	1593
18	Ledenoxide-(I)	48.64	1.19	1605
19	Aromadendrene epoxide-(II)	49.40	4.84	1625
20	Calarenepoxide	51.36	3.89	1674
21	Kauran-18-al	54.28	1.51	1751
	**Identified**		**98.23**	
	**Non terpenoids**		**11.7**	
	**terpenoids**		**86.53**	
	**Monoterpene hydrocarbones**		**0.6**	
	**sesquiterpene hydrocarbones**		**10.8**	
	**oxygenated sesquiterpene**		**75.3**	

### Identification of compounds in n-hex extract

Data on the compounds identified in the *n*-Hex extract of *F. vulgaris* obtained by injection into the GC-MS are plotted in Table 5.

**Table 5:** Characterization of volatile compounds identified in *n*-Hex extract

No	Name	Rt	Area%	KI
1	Hexene	5.062	5.72	585
2	Octanoic Acid	16.688	3.31	1157
3	Dodecane	18.031	1.08	1200
4	Tetradecane	23.923	17.64	1401
5	isospathulenol	28.436	9.94	1571
6	(−)-Spathulenol	28.652	16.91	1580
7	SALVIAL-4 (14)-EN-1-ONE	29.017	1.28	1594
8	2,6,10-Trimethylpentadecane	29.183	10.64	1600
9	Widdrol	30.267	1.66	1645
10	Himbaccol	30.526	1.18	1656
11	1-[1-Methoxy-3,3-dimethyl-2-(3-methylbuta-1,3-dienyl)cyclopentyl]ethanone	31.356	1.18	1690
12	Limonene dioxide	31.581	3.09	1700
13	9,10Dimethyltricyclo [4.2.1.1 (2,5)] decane-9, 10-diol	32.18	1.16	1725
14	Isoaromadendrene epoxide	33.122	3.62	1766
15	Octadecane	33.896	3	1800
16	2-Butenal, 2-methyl-4-(2,6,6-trimethyl-1-cyclohexen-1-yl)-	34.397	4.76	1823
17	Hexahydrofarnesyl acetone	34.61	1.16	1833
18	3,7,11,15-Tetramethyl-2-hexadecen-1-ol	34.741	1.14	1839
19	Hexadecanoic acid	36.997	6.22	1944
20	Hexadecanoic acid, ethyl ester	37.695	2.16	1978
	**Identified**		**96.85**	
	**Non-terpenoids**		**48.97**	
	**terpenoid**		**47.88**	
	**oxygenated monoterpene**		**3.09**	
	**oxygenated sesquiterpene**		**29.69**	
	**oxygenated diterpene**		**1.16**	

### Identification of compounds in 40% VLC fraction of n-hex extract

The data on the compounds identified in the 40% VLC fraction of *n*-Hex extract of *F. vulgaris* obtained by injection into the GC-MS are presented in Table 6.

**Table 6:** Specifications of compounds identified in 40% VLC fraction of *n*-Hex extract

No	Name	Rt	Area%	KI
1	Octanoic Acid	16.27	3.31	1157
2	trans-Caryophyllene	18.30	2.30	1422
3	germacrene d	20.40	2.15	1480
4	caryophyllene oxide	23.20	14.25	1545
5	(−)-Spathulenol	27.68	20.4	1580
6	Widdrol	30.67	6.70	1645
7	Limonene dioxide	37.87	4.20	1700
8	Octadecane	42.61	5	1800
9	Hexadecanoic acid	46.52	11.20	1944
10	Hexadecanoic acid, ethyl ester	47.60	1.12	1978
	**Identified**		**70.47**	
	**Non-terpenoids**		**22.72**	
	**terpenoid**		**47.75**	
	**monoterpene**		**4.20**	
	**sesquiterpene**		**43.55**	

### Evaluation of cytotoxicity of volatile oil

The results of cytotoxicity of *F. vulgaris* volatile oil on SW-872 cancer cells in 24-h treatment are 3.78 ± 0.93 which found that the highest toxicity effect occurred on SW-872 cell line.

## Discussion

According to the World Health Organization (WHO), 9.6 million deaths in 2018 worldwide will be due to various types of cancer, and the number of these deaths is projected to reach more than 11 million per year by 2030 [33]. Today, the world is facing a high prevalence of cancer, which is the second leading cause of death after heart disease. Understanding the important mechanisms involved in causing cancer is important for advancing therapies for the treatment of neoplasms [34]. Chemotherapy drugs should ideally have a specific cytotoxic effect on neoplastic cancer cells; however, in reality this treatment leads to some systemic toxicity to the individual [35]. Apoptosis, the best programmed cell death, is involved in controlling the number of normal cells and their proliferation as part of the natural development process [36].

75–80% of the world’s populations, especially in developing countries, use herbal medicines to treat diseases. This is because they believe that herbal medicines, in addition to being cheap and available, have fewer side effects than chemical drugs. Many common medicines are derived from plant sources. In the past, the basis of many drugs was herbal, including aspirin (bark willow), digoxin (fox glove) and morphine (opium poppy), etc. [37]. Various studies have shown that plant compounds play an important role in both the prevention and treatment of cancers. These compounds work by different mechanisms; however, the induction of apoptosis is a common point of many of these compounds [38]. The effect of coumarins on the cytotoxic effect of members of the Apicaceae family has also been proven in studies [39–41].

Many studies have shown that different plants with different medicinal compounds have anti-cancer effects. For example, Bhandari et al. showed that different IC50 levels of *Allium wallichii* extract were effective on prostate cancer cell lines (69.69%), breast (55.29%), and cervix (46.51%) [42]. Afsar et al. evaluated the anti-cancer activity of aerial parts of *Acacia hydaspica* R. Parker. They found that IC50 of about 29.9 ± 0.909 μg/ml was effective on the MDA-MB-361 cancer cell line and IC50 of about 39.5 ± 0.872 μg/ml on the HCC-38 cell line [43]. Lai et al. evaluated the anti-cancer activity of *Blechnum Orientale Linn* extract and showed that at IC50 values of about 27.5–42.8 μg/ml, it has cytotoxic activity against HT-29 cell line. The phytochemical analysis also showed the presence of compounds such as flavonoids, terpenoids, and tannins [44]. Using *Cassia angustifolia* Vahl extract, Ahmed and his colleagues were able to obtain IC50 values of about 4.0 μg/μL, 5.45 μg/μL, and 7.28 μg/μL against MCF-7, Hela, and HepG2 cancer cell lines, respectively [45]. Chaudhary et al. used different fractions of *Nardostachys jatamansi* DC to study its anti-cancer activity and concluded that the extract of this plant on breast cancer cell lines including MCF-7 (IC50 about 58.01 ± 6.1 μg/ml) and MDA-MB-231 (IC50 about 23.83 ± 0.69 μg/ml) was effective. In addition, they showed that the highest anti-cancer activity was attributed to the presence of compounds such as Lupeol and beta-sitosterol [46]. The significant antioxidant effect of *F. vulgaris* due to the presence of compounds such as alkaloids, anthraquinones, flavonoids, phenols, saponins, steroids and tannins has already been investigated [24]. Antioxidant compounds such as spathulenol and carvacrol have also been identified as the most essential oils of the plant [25]. In the present study, the cytotoxic effects of *n*-Hex, DCM and MeOH extracts of *F. vulgaris* on SW-872 (skin cancer) and HFF-2 cell lines were investigated for the first time. In this regard, at the beginning of the work, cytotoxicity and IC_50_ values ​​obtained from the treatment of these cells with different extracts were investigated. According to evaluate the cytotoxicity of the plant on human liposarcoma cells (SW-872), MTT assay was performed with three extracts of *n*-Hex, DCM and methanol. MeOH extract had no notable effect on cell growth and *n*-Hex extract had the most cytotoxic effect compared to DMSO control (*p* < 0.0001) and DCM extract, at time 24 (*p* < 0.001) and 48 h (*P* < 0.01). Flow cytometric results of this extract showed that the mechanism of cell death induced by it was both apoptosis and necrosis. In order to further investigate and isolate the apoptosis-inducing compounds, *n*-Hex extract was selected for further study. The results of cytotoxicity of *n*-Hex extract fractions on SW-872 show that the anticancer effect of all fractions in 24- and 48-h treatment is significantly different from the control (*p* < 0.0001). The 40 and 60% fractions have the best IC_50_ values among the samples so that the cytotoxic effect of these two samples is not significantly different from each other. 40% fraction showed the highest amount of cytotoxicity which is significantly different from the effect of other fractions (*p* < 0.05). According to Table 2, although all *n*-Hex VLC fractions inhibited the cancer cell lines compared to the control group significantly, IC50 amount of 40% VLC fraction was low. Furthermore, as Table 3 illustrates, the toxic effect of 40% VLC fraction on normal cell lines has the lowest as well. It means that it could be the best candidate for more fractionation and more isolation of pure compounds with low side effects. Moreover, based on Figs. 3, 40% VLC fraction of *n*-Hex extract has the highest induction of apoptosis on SW-872 cells and has a suitable cell death mechanism. In addition, according to Table 3, the toxic effect of 60% VLC fraction on normal cells is more than cancer cells which means that this fraction may have most side effects via induction of necrosis on normal cells.

The essential oil components of this plant, which were collected from Moghan region in spring, are abundant in the category of compounds with the structure of oxygenated sesquiterpene (75.3%), sesquiterpene hydrocarbon terpenes (10.8%), hydrocarbon monoterpenes (0.6%) were found and the total terpene content of essential oil was 86.53%. The results also showed that spathulenol (33.8%) and caryophyllene oxide (18.3%) are the most abundant volatile compounds of *F. vulgaris,* respectively. The yield of volatile oil was determined as 0.206% by volume/weight. Caryophyllene oxide has a considerable cytotoxic effect on different cancer cells that is dose and time-dependent. The best reported IC_50_ value of caryophyllene oxide isolated from *Psidium cattleianum* is 3.95 ± 0.23 μM [47]. Spathulenol has also been suggested as a good candidate for the treatment of drug resistance in cancer treatment [48]. Therefore, the effect of remarkable cytotoxicity (*p* < 0.0001) of *F. vulgaris* essential oil on cancer cells can be considered due to the presence of valuable anti-cancer compounds such as caryophyllene oxide and spathulenol. Another species of this plant that grows in Iran is *F. falcariodes*. The results of a study conducted by Masoudi et al. on the plant identified 24 compounds that make up 97.6% of the total volatile oil of the plant; among these compounds, germacrene B (67.9%) is the main compound of this plant [26].

The most probable compounds identified by GC-MS method of *n*-Hex extract are non-terpenoid compounds, most of which include tetradecane (17.64%), spathulenol (16.91%), trimethyl pentaacetane (10.64%), isospathulenol (9.94%) and hexadecanoic acid (6.22%), respectively and 40% VLC fraction of *n*-Hex extract has the highest terpenoid compounds such as spathulenol (20.4%), caryophyllene oxide (14.25%) and hexadecanoic acid (11.2%). In this regard, many studies have proven the cytotoxic role of compounds in plant essential oils. For example, Oliveira et al. Reported the cytotoxic effects of active volatile oil of several plants with medicinal properties on cancer cell lines such as tumor cell lines murine melanoma (B16F10), human colon carcinoma (HT29), human breast adenocarcinoma (MCF-7), human cervical adenocarcinoma (HeLa), human hepatocellular liver carcinoma (HepG2), human glioblastoma (MO59J, U343, and U251), and Normal hamster lung fibroblasts (V79 cells) were studied. They concluded that compounds such as spathulenol and caryophyllene have significant cytotoxic effects as part of the active compounds present in the plant [49]. In another study by Mellado et al., The effect of essential oils of *Ephedra chilensis* extract on a variety of cancer cell lines was investigated. Using GC-MS analysis, 2.4% of the total compounds in dichloromethane extract are tetradcanoic acid and hexadecanoic acid, which have significant cytotoxic effects on prostate and breast cell lines [50].

## Conclusion

The results showed that 40% fraction of *n*-Hex extract and *F. vulgaris* essential oil on SW-872 cells have a significant cytotoxic effect. The cytotoxicity of the fraction is completely dose-dependent and significantly (*p* < 0.0001) higher than the control group. Furthermore, the mechanism of cell death in 40% *n*-Hex fraction is predominantly through induction of apoptosis, which can be concluded that apoptosis-inducing compounds are probably in the effective fractions of *n*-Hex extract. It is worth mentioning that the 40% *n*-Hex fraction has a selective effect on SW-872 cancer cells compared to normal non-cancer cells. The appropriate properties mentioned in the effective samples have raised great hopes for the purification of effective anti-cancer compounds with minimal side effects from *F. vulgaris* in future works.

## Data Availability

The datasets used and/or analyzed during the current study are available from the corresponding author on reasonable request.

## References

[1] Organization WH. cancer. December 2018.

[2] Mathur P, Sathishkumar K, Chaturvedi M, Das P, Sudarshan KL, Santhappan S (2020). Cancer statistics, 2020: report from national cancer registry programme, India. JCO Global Oncol.

[3] Saunders FR, Wallace HM (2010). On the natural chemoprevention of cancer. Plant Physiol Biochem.

[4] Boopathy NS, Kathiresan K. Anticancer drugs from marine flora: an overview (online). J Oncol. 2010;2010:214186 10.1155/2010/214186 PMC3065217 21461373

[5] Prakash O, Kumar A, Kumar P (2013). Anticancer potential of plants and natural products. Am J Pharmacol Sci.

[6] Weli AM, JR AL-H, Al-Mjrafi JM, Alnaaimi JR, Hossain MA, Saeed S (2014). Effect of different polarities leaves crude extracts of Omani juniperus excels on antioxidant, antimicrobial and cytotoxic activities and their biochemical screening. Asian Pacific J Reprod.

[7] Kang JX, Liu J, Wang J, He C, Li FP (2005). The extract of huanglian, a medicinal herb, induces cell growth arrest and apoptosis by upregulation of interferon-β and TNF-α in human breast cancer cells. Carcinogenesis..

[8] Mohammadi A, Mansoori B, Aghapour M, Baradaran PC, Shajari N, Davudian S (2016). The herbal medicine Utrica dioica inhibits proliferation of colorectal cancer cell line by inducing apoptosis and arrest at the G2/M phase. J Gastrointest Cancer.

[9] Mohammadi A, Mansoori B, Goldar S, Shanehbandi D, Mohammadnejad L, Baghbani E (2016). Effects of Urtica dioica dichloromethane extract on cell apoptosis and related gene expression in human breast cancer cell line (MDA-MB-468). Cell Mol Biol.

[10] Portt L, Norman G, Clapp C, Greenwood M, Greenwood MT (2011). Anti-apoptosis and cell survival: a review. Biochimica et Biophysica Acta (BBA)-molecular. Cell Res.

[11] Sun S-Y, Hail N, Lotan R (2004). Apoptosis as a novel target for cancer chemoprevention. J Natl Cancer Inst.

[12] Thangam R, Sathuvan M, Poongodi A, Suresh V, Pazhanichamy K, Sivasubramanian S (2014). Activation of intrinsic apoptotic signaling pathway in cancer cells by Cymbopogon citratus polysaccharide fractions. Carbohydr Polym.

[13] Felipe KB, Kviecinski MR, da Silva FO, Bücker NF, Farias MS, Castro LSEPW (2014). Inhibition of tumor proliferation associated with cell cycle arrest caused by extract and fraction from Casearia sylvestris (Salicaceae). J Ethnopharmacol.

[14] Moshafi MH, Mehrabani M, Mahdikhani S, Saffari F (2015). Antibacterial effects of different fractions of extract of Falcaria vulgaris bernh fruits and bioautography of its effective fraction. Med J Tabriz Univ Med Sci Health Serv.

[15] Shakibaie D, Pasharavesh L, Khoshboo S, Kaboodi B (2007). The effect of the “Falcaria vulgaris” on deep skin wound remodeling time and skin tension power in rats. J Kermanshah Univ Med Sci.

[16] Choobkar N (2015). Effect of using Falcaria vulgaris on skin wound healing and immune response of common carp (Cyprinus carpio). Vet Clin Pathol Q Sci J.

[17] Yadegari M, Khazaei M, Ghorbani R, Rezae M, Izadi B, Sheikholeslam A. Wound Healing Effect of Falcaria Vulgaris’ Leaves on Aspirin Induced Gastric Ulcer in Rats. J Kermanshah Univ Med Sci. 2006;10(3).

[18] Gadekar R, Singour P, Chaurasiya P, Pawar R, Patil U (2010). A potential of some medicinal plants as an antiulcer agents. Pharmacogn Rev.

[19] Khazaei M, Salehi H (2006). Protective effect of of *FALCARIA Vulgaris* extract on ethanol induced gastric ulcer in rat.

[20] Yadegari M, Khazaei M, Hamzavi Y, Toloei AR (2011). Antifertility effects of Falcaria vulgaris in female rat. J Arak Univ Med Sci.

[21] Abolhassanzadeh Z, Aflaki E, Yousefi G, Mohagheghzadeh A. Medicinal plants for joint pain in traditional Persian medicine. Trends in Pharmaceutical Sciences. 2016 Jun 1;2(2).

[22] Rouhi-Boroujeni H, Asadi-Samani M, Moradi M-T (2016). A review of the medicinal plants effective on headache based on the ethnobotanical documents of Iran. Pharm Lett.

[23] Goudini A, Shakibaei D (2007). Effect of *FALCARIA Vulgaris* extract on Isolated rat heart.

[24] Jaberian H, Piri K, Nazari J (2013). Phytochemical composition and in vitro antimicrobial and antioxidant activities of some medicinal plants. Food Chem.

[25] KhanAhmadi M, Shahrezaei F (2007). Study on chemical constituents of the essential oil of Falcaria vulgaris Bernh. J Med Plants.

[26] Masoudi S, Ameri N, Rustaiyan A, Moradalizadeh M, Azar PA (2005). Volatile constituents of three Umbelliferae herbs: Azilia eryngioedes (Pau) hedge et Lamond, laser trilobum (L.) Borkh. And Falcaria falcarioides (Bornm. Et Wolff) growing wild in Iran. J Essent Oil Res.

[27] Shafaghat A (2011). Volatile oil constituents and antibacterial activity of different parts of Falcaria vulgaris Bernh. Growing wild in two localities from Iran. Nat Prod Res.

[28] Shafaghat A (2010). Free radical scavenging and antibacterial activities, and GC/MS analysis of essential oils from different parts of Falcaria vulgaris from two regions. Nat Prod Commun.

[29] Assadi M, Ramak Maassoumi AA, Khatamsaz M. Flora of Iran. Tehran: Ministry of Agriculture, Islamic Republic of Iran; 1989.

[30] Maurya A, Kalani K, Verma SC, Singh R, Srivastava A (2018). Vacuum liquid chromatography: simple, efficient and versatile separation technique for natural products. Org Med Chem IJ.

[31] Targett NM, Kilcoyne J, Green B (1979). Vacuum liquid chromatography: an alternative to common chromatographic methods. J Organic Chem.

[32] Wesołowska A, Jadczak P, Kulpa D, Przewodowski W (2019). Gas chromatography-mass spectrometry (GC-MS) analysis of essential oils from AgNPs and AuNPs elicited Lavandula angustifolia in vitro cultures. Molecules..

[33] World Health Organization. Cancer burden rise to 18.1 million new cases and 9.6 million cancer deaths in 2018. Int Agency Res Cancer. 2018;2018:3.

[34] HemaIswarya S, Doble M (2006). Potential synergism of natural products in the treatment of cancer. Phytother Res.

[35] Johnstone RW, Ruefli AA, Smyth MJ (2000). Multiple physiological functions for multidrug transporter P-glycoprotein?. Trends Biochem Sci.

[36] Xu G, Shi Y (2007). Apoptosis signaling pathways and lymphocyte homeostasis. Cell Res.

[37] Pal SK, Shukla Y (2003). Herbal medicine: current status and the future. Asian Pac J Cancer Prev.

[38] Najda A, Dyduch J, Świca K, Kapłan M, Papliński R, Sachadyn-Król M (2015). Identification and profile of Furanocoumarins from the ribbed celery (Apium Graveolens L Var. Dulce mill./Pers.). Food science and technology. Research..

[39] Shokoohinia Y, Sajjadi S-E, Gholamzadeh S, Fattahi A, Behbahani M (2014). Antiviral and cytotoxic evaluation of coumarins from Prangos ferulacea. Pharm Biol.

[40] Orhan IE, Senol FS, Shekfeh S, Skalicka-Wozniak K, Banoglu E (2017). Pteryxin-a promising butyrylcholinesterase-inhibiting coumarin derivative from Mutellina purpurea. Food Chem Toxicol.

[41] Beltagy AM, Beltagy DM (2015). Chemical composition of Ammi visnaga L. and new cytotoxic activity of its constituents khellin and visnagin. J Pharm Sci Res.

[42] Bhandari J, Muhammad B, Thapa P, Shrestha BG (2017). Study of phytochemical, anti-microbial, anti-oxidant, and anti-cancer properties of Allium wallichii. BMC Complement Altern Med.

[43] Afsar T, Razak S, Khan MR, Mawash S, Almajwal A, Shabir M (2016). Evaluation of antioxidant, anti-hemolytic and anticancer activity of various solvent extracts of Acacia hydaspica R. Parker aerial parts. BMC Complement Altern Med.

[44] Lai HY, Lim YY, Kim KH (2010). Blechnum orientale Linn-a fern with potential as antioxidant, anticancer and antibacterial agent. BMC Complement Altern Med.

[45] Ahmed SI, Hayat MQ, Tahir M, Mansoor Q, Ismail M, Keck K (2016). Pharmacologically active flavonoids from the anticancer, antioxidant and antimicrobial extracts of Cassia angustifolia Vahl. BMC Complement Altern Med.

[46] Chaudhary S, Chandrashekar KS, Pai KSR, Setty MM, Devkar RA, Reddy ND (2015). Evaluation of antioxidant and anticancer activity of extract and fractions of Nardostachys jatamansi DC in breast carcinoma. BMC Complement Altern Med.

[47] Jun NJ, Mosaddik A, Moon JY, Ki-Chang J, Dong-Sun L, Ahn KS (2011). Cytotoxic activity of [beta]-Caryophyllene oxide isolated from jeju guava (Psidium cattleianum sabine) leaf. Records Nat Prod.

[48] Martins A, Hajdú Z, Vasas A, Csupor-Löffler B, Molnár J, Hohmann J (2010). Spathulenol inhibit the human ABCB1 efflux pump. Planta Med.

[49] de Oliveira PF, Alves JM, Damasceno JL, Oliveira RAM, Dias HJ, Crotti AEM (2015). Cytotoxicity screening of essential oils in cancer cell lines. Rev Bras.

[50] Mellado M, Soto M, Madrid A, Montenegro I, Jara-Gutiérrez C, Villena J (2019). In vitro antioxidant and antiproliferative effect of the extracts of Ephedra chilensis K Presl aerial parts. BMC Complement Altern Med.

